# Law, Ethics, Religion, and Clinical Translation in the 21^st^ Century – A Discussion with Pete Coffey

**DOI:** 10.1002/stem.414

**Published:** 2010-03-22

**Authors:** Majlinda Lako, Alan Trounson, Susan Daher

## About Professor Pete Coffey

Prof. Coffey received a B.Sc. in Experimental Psychology from the University of Sussex in 1986 and then went on to earn his D.Phil in Experimental Psychology from the University of Oxford in 1995. On completing his doctoral work, Prof. Coffey joined the faculty at Oxford University in the Department of Experimental Psychology. In 1989 he was awarded a Robert and Joan Case Royal Society Research Fellowship, and at the same time moved to the University of Sheffield, subsequently becoming a lecturer and senior lecturer before moving to London in 2004. Prof. Coffey is now Chair of Cellular Therapies and Visual Sciences at the Institute of Ophthalmology at University College London. He is also Research Head of the Department of Ocular Biology and Therapeutics.

Among his many achievements, he has done seminal work on retinal transplantation, he is the principle author and co-author of two landmark papers demonstrating the use of human cells to halt visual deterioration in models of age-related macular degeneration, he developed the first transplant strategy to treat inherited macular degeneration, and he has developed a cell-based therapy for age-related macular degeneration. He also helped launch, and is now the Director of, the London Project to Cure Blindness.

## “People Have Been Investigating Stem Cells to Repopulate the Cells of the Cornea… for the Last 10 Years.”

Even before stem cells began to make their mark on research and therapeutics, Prof. Coffey was working on cell therapies to replace dying cells in the eye. He was studying techniques based on the use of adult cells that had been either taken from biopsies or that had been donated, which were then immortalized and scaled up. But, explains Prof Coffey, “with the emergence of several breakthroughs in stem cell science, it became clear very quickly that this new technology was where the field should be headed. Stem cell technology promised the new ability to take a fresh cell and use this as the raw material to actually produce other new, fresh cells.”

Research in Prof. Coffey's lab now focuses on stem cell therapeutics, along three main lines. “In my laboratory, we are interested in using stem cells to understand and treat diseases of the eyes, and in particular, disease affecting the back of the eye, or the neural retina, as well as the cells and vasculature that support the neural retina. To do this, we are differentiating human ESCs into the types of cells that have been lost due to disease.” Interestingly, the use of stem cells in ophthalmology is not new. “For the last 10 years people have been looking at stem cells to repopulate the cells of the cornea, which may have been damaged due to acid or alkali burns, or because of genetic disease. So the front of the eye has already been served quite well by the use of stem cells. But now, new therapies are beginning to emerge for the back of the eye.”

“We are also investigating the possibility of using bioengineered scaffolds so that we can put the cells back into patients in a much more structured way; instead of just injecting the cells, we can actually form patches of cells which are properly differentiated and have all the characteristics of the cells which have died and which we would like to replace. A third line of investigation in my lab is the newer technology of iPS cells. That is, being able to take a somatic cell, typically something like a skin cell, and turn it into a stem cell, using the knowledge we've gained from our studies of hESCs.”

## “We Are Aiming to Begin Trials [for Macular Degeneration] in 2011.”

“I think that the first use of stem cells in the back of the eye will be the use of stem cells to repopulate the retinal pigment epithelial cells, which support the neural retina, and are intimately involved with the blood supply at the back of the eye. One disease that shows promise to be treated with this type of stem cell therapy is age related macular degeneration, which results in a loss of vision in the central visual filed due to damage to the retina. This is an enormous clinical problem, and there aren't any good therapies available at the moment for the dry form of the disease. We are beginning to undertake early stage trials, phase I and phase II, using stem cell to treat macular degeneration. We're differentiating hESCs into retinal pigmented epithelia cells, and then putting these back into patients with age related macular degeneration where cells have died. Right now, we are in the throws of going through the regulatory processes required to undertake these trials, as well as producing these cells to clinical grade standards, and we aim to begin these trials in 2011.”

“Another major disease in terms of the size of the clinical need is diabetic retinopathy, which is probably the biggest ophthalmological problem in the working population. Here, we need to find a way of replacing the vasculature that is affected in this disease. I also think that there is the possibility of eventually replacing damaged photoreceptor cells, to help conditions such as retinal pigmentosa.” There are also researchers looking at the possibility of using stem cells in glaucoma, a disease in which there is loss of retinal ganglion cells, leading to damage of the optic nerve and loss of vision. “Although there are some people looking at replacing these cells, the major problem is that these cells travel a very long distance to connect to the brain, and in addition, there are a number of structures to which they must connect. So I think that glaucoma treatment will likely be more about trying to protect these cells and keep them from dying rather than replacing them.”

**Figure:**
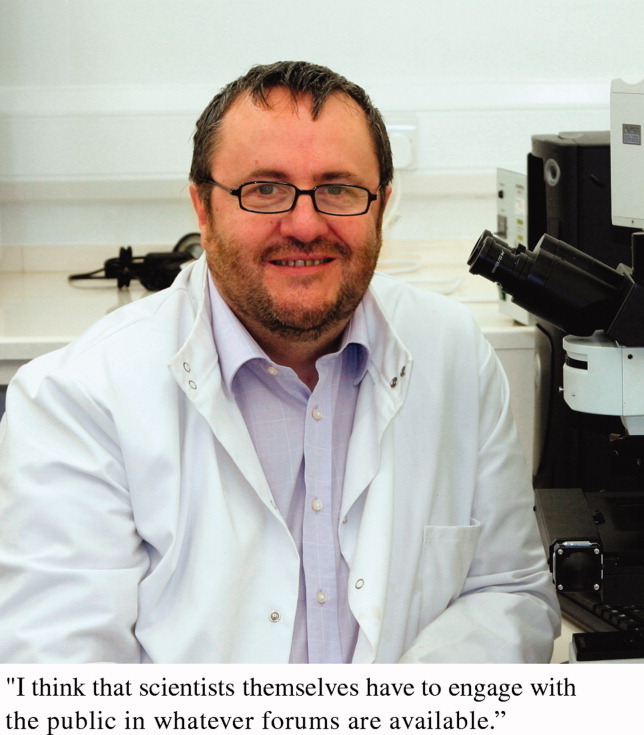
Photo. Pete Coffey, PhD., Institute of Ophthamology, University College London.

## “The Possibilities of Using (iPS Cells) to Understand Disease Processes and to Identify New Therapeutic Targets are Immense.”

“I think one of the most important developments in stem cell science is, and will continue to be, our ability to properly differentiate the cells. We need to be able to get the specific eye cells that we need, such as the retinal pigment epithelial cells or photoreceptors, to enable at least the possibility of considering these as a source of therapeutic value in terms of cell replacement. This requires a lot of knowledge surrounding the developmental biology of the eye, in combination with the biology of stem cells.”

“This is why there is real excitement now about the new technology of iPS cells. While this technology is still in its infancy, and probably at the moment still isn't positioned to be used as a cell replacement therapy in its own right (although I do believe that will happen, and probably very soon), they provide immense possibilities to study development and disease processes. They provide us with the phenomenal possibility of actually taking samples from specific patients with a disease and then producing in the Petri dish the very cells which are affected by the disease; these can then be used to study disease pathogenesis and develop bioassays. We can then probe the very cells which are being diseased in those patients, and look at potential therapeutic targets. I think this will really take off and evolve quite quickly, and will be very beneficial for the field of ophthalmology, where there are several known genetic defects which lead to disease.”

“At the moment, hESCs definitely have more potential to treat many people. Because in this case, potentially, ‘one will fit all’. We'll know this very soon, in 2011 when we begin our macular degeneration trials, if that's the case. So I think in terms of therapeutic value, the hESCs still have huge potential and probably will be the major type of stem cell going into the clinic in the beginning. I think there is still an issue about whether iPS cells can really be seen as a technology for therapeutics. It would be very expensive to produce a cell from a specific patient and then go through all of the quality assessment and scaling up that would be needed to go into the clinic. I think that there are still a number of significant issues that need to be addressed before we are able to consider using iPS cells as therapeutics. However, as I said, the possibility of using them to understand disease processes and identify new therapeutic targets is immense.”

## Proactive Legislation “is Necessary to Ensure the Safety of Patients.”

“One of the biggest obstacles that needs to be overcome in order to use stem cells therapeutically is to ensure that the cells are safe. And they need to be safe in two different ways. First, we must ensure that the cells do not possess the ability to form teratomas, and second, we must ensure that the immunological responses that could be triggered by these cells are not going to be deleterious. While the possibility of teratomas, of course, needs to be excluded completely, in terms of the immunological response, it may be possible to manage this by some type of therapeutic intervention, for example, by immunosupression. But these problems are beginning to be addressed; we now have studies identifying what things definitely need to be targeted to make sure there is no ongoing proliferation in the cells which could lead to teratomas, and it is also now clear that some of these cells don't express a major immunogenicity component, so immune suppression may not be necessary.”

“The other big obstacle to using stem cells therapeutically is the scaling up of the cells, to obtain the large number of cells that you need for each patient, and how to ensure that they are still produced to clinical grade standards and still meet quality control levels. To undertake early trials in phase I and phase II, only a limited number of cells are needed, because only a few patients, say 10 or so, will be enrolled in the trials. But there are hundreds of thousands of patients in the clinical population in general, and so many more cells would have to be produced to meet the needs of these patients if these cells were to be used clinically.”

Dr. Coffey believes that the legislation regarding stem cells in the UK will continue to be helpful in getting stem cell therapies into the clinic. “In the UK, the legislation surrounding the use of stem cell is very positive, and proactive I think. Stem cell research and therapeutics is highly regulated, and there are a number of organizations that are involved in the process from the very onset, including the Human Fertility and Embryology Authority, the Human Tissue Authority, the Medicines and Healthcare Products Regulatory Agency, as well as other ethics committees such as the Gene Therapy Advisory Commission in Europe. So although there is legislation, I would say that it is very proactive, and while it is quite a process to go through, it is helping us to ultimately reach the clinic with these treatments, and I also feel is it necessary to ensure the safety of patients.”

“There can be some problems involved in collaborating with researchers in countries that do not have the same types of legislation. For instance, until recently in the US no federal funds could be used for human embryonic research, unless the research used a few specifically approved cell lines, although that has changed with the new administration. And some states have on their own taken a more proactive stance to stem cell legislation. For instance California took a stance early on to be very proactive in the use of hESCs, and made a very bold statement which the tax payers themselves are paying for. This lead to the establishment of the California Institute for Regenerative Medicine (CIRM), where I currently have productive collaborations. However, there are some countries where stem cells research is without doubt very difficult, particularly for labs trying to get funding for hESC research, where investigators still have to spend a lot of time just battling for basic funding to do the research.”

## The Pathway to Clinical

Dr. Coffey believes that over the next 10 years the most important aspect of stem cell research will be the pathway to clinic. “If this technology is really going to step up to the mark, then we really do need to fully understand all the issues in this pathway to clinic, which includes safety and toxicity issues, as well as regulatory issues - we need to know what exactly the regulatory authorities will expect. And again, if this is to be seen as a viable therapeutic, we need to address the issues of scaling up the process, to be able to meet the clinical needs throughout the world.”

“As we get closer to using stem cell as therapies in clinics, we do hear worries from the general public – and many are misconceptions. I think one is the misconception about the biology of stem cells. There is this idea that no matter what the disease is, stem cells will cure it for you. There has to be the understanding that stem cells are not ‘magical’ cells; they still function within the bounds of biology, so we can't just expect them to magically cure people, that's not the way it works. I think another major public misconception is that actually getting the stem cells is the problem, but it's not. We do have the stem cells, the problems are around how we turn these stem cells into the cells we need to benefit that clinical population.”

“I think we have to be honest with the public in terms of the number of applications that there will be initially – there will be only a few – possibly age related macular degeneration, and maybe some aspects of heart disease and diabetes, for instance. But the type of press releases that are often seen around, for example, Alzheimer disease or Parkinson's disease, may be premature. I think that neural replacement treatments will be a lot more complex because they involve very complex neural systems, in which the cells will have to interact properly with each other.”

“Another misconception in the public is that we are going to have to go back continually for the blastocysts – the eggs from which we derive the hESCs, but that's not necessary. To give one example, we're trying now to scale up stem cells for use in macular degeneration, a clinical population that includes millions of people globally, but we do not need to keep going back to fertilized eggs. We are using only one hESC line, and that should be able to scale up to produce enough cells for that whole clinical population”.

“Scientists could better educate the public by discussions, or have meetings where everyone can discuss the whole agenda around stem cells, such as what they are and how they are used. Just recently I did a presentation at the Wellcome Collection Gallery in London, and next week I will be going up to the north of England to visit a local macular degeneration society, to discuss issues around stem cells. I think that scientists themselves have to engage with the public in whatever forums are available.”

“I am also Director of the London Project to Cure Blindness. Back in April 2007, following a major donation from an American philanthropist, we set up this Project specifically to help take a cell therapy for age related macular degeneration from the lab into the clinic. The whole goal was to start to scale up the whole process from the beginning, and to bring on board from the very start of the project not just basic scientists but clinical scientists and engineers, and people involved in the whole process of getting treatments into the clinic. Usually, research like this is done in serial; that is, you do a bit of basic research, then you do a bit preclinical work, then you look at what may be the clinical issues. Doing this serially takes a lot of time and at each stage there is also the problem of finding funding. But this substantial donation allowed a very cross-disciplinary research group to work together from the very start, to try to power very quickly the translation from the basic science to the clinic, and I think that is a valuable research model.”

